# Effect of Membrane Components of Transverse Forces on Magnitudes of Total Transverse Forces in the Nonlinear Stability of Plate Structures

**DOI:** 10.3390/ma13225262

**Published:** 2020-11-20

**Authors:** Zbigniew Kołakowski, Jacek Jankowski

**Affiliations:** Department of Strength of Materials, Faculty of Mechanical Engineering, Lodz University of Technology, Stefanowskiego 1/15, PL-90-924 Lodz, Poland; jacek.jankowski@p.lodz.pl

**Keywords:** nonlinear stability, square plate, shear forces, components of transverse forces in bending, membrane components of transverse forces, 4 methods (CPT, FSDT, S-FSDT, FEM)

## Abstract

For an isotropic square plate subject to unidirectional compression in the postbuckling state, components of transverse forces in bending, membrane transverse components and total components of transverse forces were determined within the first-order shear deformation theory (FSDT), the simple first-order shear deformation theory (S-FSDT), the classical plate theory (CPT) and the finite element method (FEM). Special attention was drawn to membrane components of transverse forces, which are expressed with the same formulas for the first three theories and do not depend on membrane deformations. These components are nonlinearly dependent on the plate deflection. The magnitudes of components of transverse forces for the four theories under consideration were compared.

## 1. Introduction

In the mid-20th century, Reissner [1,2] presented a plate theory accounting for the transverse shear deformation effect. This is a stress-based approach. Mindlin [3] offered a theory based on a displacement approach, where transverse shear stress was assumed to be the same through the plate thickness, and the shear correction factor *k*^2^ (the so-called Mindlin correction factor) appeared. For transversely inextensible plates and *k*^2^ = 5/6, values of stresses are equal in the Reissner and Mindlin plate theories [4]. The theories are characterised by an equivalent approximation degree known as the Reissner–Mindlin plate theory. Their comparison is discussed in [5,6,7,8], etc. Theoretical considerations can be found in [9,10,11,12,13,14,15] for higher-order shear deformation theories as well.

In [4,5], the equations of Reissner and Mindlin plates, including the parameter, which allows for an interpretation of these theories for transversally inextensible plates, were derived. In [6], for Reissner, Mindlin and Reddy plate models, a solution to the rectangular transverse plate sinusoidally loaded and freely supported along all the edges is given. Vibrations in the plate–beam system, in which the Reissner–Mindlin plate model related to the Timoshenko beam model was applied, were analysed in [7]. In [11,16], it is shown that when the plate thickness is around zero, the solution for the Reissner–Mindlin plate becomes close to the solution within the Kirchhoff–Love plate theory (the so-called classical plate theory (CPT)). In [17], formulations of the mixed finite element were given based on the mechanism of the shear locking phenomenon and the general variation method for the Reissner theory, including the Lagrange multiplier method. An extensive literature survey devoted to the two primary plate theories, i.e., the Kirchhoff plate theory and the Reissner–Mindlin plate theory, is found in [18]. The main purpose of [18] was to present a history of refinement in the Reissner–Mindlin theory, showing the up-to-date state of knowledge in this field of research.

Within the plate theories accounting for the shear deformation effect (FSDT), one can find other approaches, established, for instance, on superposition of bending and shear deflections (i.e., a two-variable refined plate theory [19,20,21,22,23,24,25,26,27,28,29,30] or a single-variable refined theory [31]).

Endo and Kimura [19] first suggested the simple first-order shear deformation theory (S-FSDT). Employing Hamilton’s principle, an alternative formula in which a deflection in bending is the primary variable instead of the angle of rotation in bending and, at the same time, some limitations on neglecting the Reissner boundary effects are imposed, was given [2,32]. In the S-FSDT, three equations in the original formulation are reduced to two and the boundary conditions are also subject to respective modifications; however, the way the system is modelled remains unaltered (for a more detailed analysis, see Appendix A.1). Moreover, in [26,27,28], when two independent variables φ and *w*_s_ are considered, two differential equations with boundary conditions are attained. In the static analysis, two differential equations are uncoupled. The boundary conditions should be uncoupled as well, which is not possible in general. By introducing a bending relationship between the quantities, differences between the Reissner and Mindlin plate theories were investigated in [8]. In [24], the first two-variable shear deformation theory (FSDT) considering in-plane rotation, which allows one to correctly predict the response of plates for arbitrary boundary conditions in the analysis of buckling and vibrations of isotropic plates, was presented.

Interesting critical remarks to the above-mentioned plate theories can be found in [32,33,34,35,36]. To consider the Reissner boundary effect, a rotary potential, which is a fast-varying solution to the boundary layer, should be applied apart from the function φ. The boundary effect covers only some boundary conditions (e.g., pure tension or contact problems). The author of [32] suggested to refer to the presented version of the theory as a modern form of the CPT.

The finite element method employs the FSDT [9,18,33,37,38,39,40,41]. The effect of shear locking in finite shell elements and a loss in accuracy was explained in terms of the occurrence of solutions to the boundary layer. A shear locking problem occurs in the FEM, as shape functions cannot approximate a fast-variable solution to the boundary layer. [33,39]. Shear locking does not cause membrane deformations. In the majority of cases, the effect of shear deformation on displacements should be considered only. Solutions to the boundary layer are neglected.

In composites widely applied nowadays, the behaviour of individual layers can be affected considerably by transverse shear deformation [42]. These materials show low shear characteristics beyond the plane, which should be accounted for in numerous analyses (e.g., [15,20,21,43]).

In the works devoted to the plate dynamics, the effect of transverse rigidity on shear deformation and the influence of rotary inertia on frequencies and modes of plate vibrations were investigated (e.g., [3,5,15,17,27,30,31]). In [44], for the plate jointly supported along the whole circumference and subjected to free vibrations, it was shown that deflections in bending and shear deformation vibrated in phase (i.e., the total deflection is equal to their sum) in the first branch, whereas in the second branch, shear deformation and bending deflections vibrate in antiphase, where the deflection in shear deformation is predominant, i.e., the total deflection is in phase along with the deflection in shear deformation.

In the literature, apart from vibrations, the stability of individual rectangular plates is analysed (e.g., [15,16]). In [22], a buckling analysis of isotropic and orthotropic plates employing a two-variable refined plate theory is presented, whereas in [43], the S-FSDT for composite plates with four unknowns is discussed. The authors do not know any works devoted to the nonlinear stability of plates accounting for the transverse shear deformation effect.

All the works under discussion refer to the cases when there are no membrane forces in the plate structure. These forces appear in thin-walled structures (where *h/a* < 0.05) for loads exceeding the critical loads, that is to say, for postbuckling equilibrium paths. In the literature known to the authors of the present paper, there is a lack of works devoted to the nonlinear stability of thin-walled plates, in which transverse shear deformation is considered.

In the classical theory of thin plates (CPT), total equivalent transverse Kirchhoff forces were introduced only in [36,45] employing the variational method as far as the literature known to the authors is concerned. In the CPT, a notion of equivalent Kirchhoff transverse forces is introduced to satisfy a proper number of boundary conditions. In the variational approach to the CPT, there is no need to introduce the notion of Kirchhoff forces. These forces “emerge themselves” from the theory in such an approach. As shown in [46], it is necessary to introduce the notion of total equivalent Kirchhoff transverse forces, which results from Stokes’ theorem concerning a change of the surface integral for the equilibrium equations into a plate circumference-oriented integral, that is to say, for the boundary conditions. In total Kirchhoff forces, two components of transverse forces appear; one of them is a derivative of internal moments, and the second is a projection of membrane forces on the transverse direction. The membrane forces also appear in other nonlinear problems, such as the deflection of thin-walled transversely loaded plates.

In the present work, the authors have decided to deal with the influence of these additional membrane components on the magnitudes of total Kirchhoff forces within the CPT for isotropic square plates subject to compression in the postbuckling state. These limitations were taken to facilitate an interpretation of the obtained results. For verification purposes, solutions to the Reissner theory (FSDT) and the Mindlin theory within the S-FSDT approach, i.e., after the introduction of two independent functions of displacements along the *z*-axis (i.e., the total lateral displacement w and the bending deflection ϕ), are presented. In the FSDT and the S-FSDT, the Reissner boundary condition was neglected. The governing equations within the three theories under consideration were derived with variational methods, allowing one to indicate two different components of transverse forces resulting from internal moments and membrane forces. For transversally inextensible plates, the membrane shear forces are independent of membrane deformation. For these three theories, the results for membrane forces and total forces were presented.

In composite materials, transverse shear deformation substantially affects the delamination of composites. In the failure criteria of composites, the impact of transverse components of membrane forces (i.e., in compression) is neglected. In the authors’ opinion, these components are predominant in the postbuckling state and should be considered in composite failure criteria. The main aim of the paper is to draw attention to the theoretical background for membrane components of transverse forces in the expressions for transverse forces in the theory of thin plates, which are not accounted for in FEM shell elements.

## 2. Formulation of the Problem

**Figure 1:**
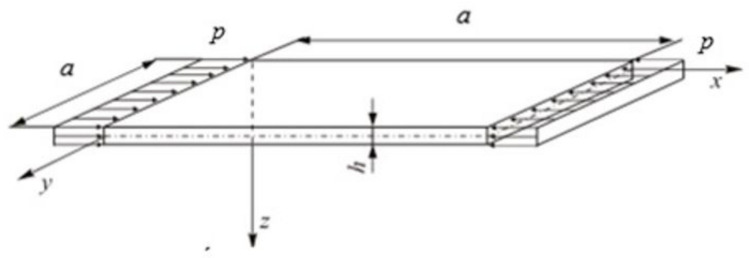
Square plate freely supported along all edges under compression.

The nonlinear stability of a square isotropic plate freely supported along the whole circumference and subject to compression along the *x*-axis (Figure 1) is analysed. The plate material is assumed to obey Hooke’s law.

In this study, for postbuckling equilibrium paths, transverse shear forces are analysed in detail for the transversally inextensible plate. The analysis is conducted within three theories of thin plates, namely the classical plate theory CPT (i.e., the Kirchhoff plate theory), the simple first-order shear deformation theory (S-FSDT) in a two-variable refined plate version and the Reissner plate theory (FSDT).

The governing equations of the three theories under consideration are presented in Appendix A.1. The equations were derived within a variational approach, which allows the equilibrium equations and the boundary conditions to be expressed explicitly. The solutions to the nonlinear problem of stability of the square plate for the three theories are presented in Appendix A.2. Instead of a system of two equations of equilibrium in the central plate plane (i.e., after an introduction of the function of Airy forces F, the system is satisfied identically), an equation of inseparability of deformations was derived (Appendix A.2.1).

According to the considerations presented in the Appendix A, transverse shear forces have two components (compare: the FSDT (A13), the S-FSDT (A24), the CPT (A33), respectively).

The first components depend on the derivatives of internal moments on the plate. Thus, the components can be referred to as transverse shear forces in bending. These forces are expressed with the following relationships: (A45) for the CPT and (A55) for the S-FSDT, correspondingly. The forces have a very similar structure. A difference lies only in the reduction factor 1/(1 + η) in (A55). Moreover, for the FSDT in (A64), a difference with respect to the S-FSDT occurs in the numerical coefficient two instead of (3-ν) for the S-FSDT. A change in the numerical coefficient results from different boundary conditions for the FSDT and the S-FSDT. A more detailed analysis can be found in the Appendix A.

The second components depend on projections of membrane transverse forces on the direction perpendicular to the central plate plane. These components can be referred to as transverse shear forces in compression. For the three theories under consideration, these forces are expressed with identical formulas (compare (A46) for the CPT, (A56) for the S-FSDT and (A65) for the FSDT in the Appendix A). It is caused by the fact that the effect of shear deformation is not accounted for, as the forces are determined on the basis of the displacement *w* and the function of Airy forces *F*.

## 3. Analysis of the Calculation Results

A detailed analysis was conducted for a steel square plate (Figure 1) of the following dimensions: *a =* 100 mm, *h =* 1 mm and the material constants: *E =* 200, GPa, ν = 0.3.

The ideal plate is supported freely along all edges and subjected to uniform compression with the stress *p* along the *x*-axis. The boundary conditions for the three theories under consideration (i.e., the FSDT, the S-FSDT and the CPT) are given in detail in the Appendix. The analytical results attained were verified with the commercial ANSYS software [47] employing the FEM (details to be found in Appendix A.2).

In the detailed analysis, the postbuckling state (or the so-called postbuckling equilibrium path) was dealt with, as only then plate deflections appear for the perfect plate. It is accompanied by the appearance of two transverse components of shear forces, that is to say, in bending and compression (the so-called membrane components).

The following index symbols are introduced in the study: C for the CPT, S for the S-FSDT, F for the FSDT, and A for ANSYS (FEM), respectively.

Firstly, for the three theories, the corresponding bifurcation loads (or the so-called critical loads), listed in detail in Table 1, were determined. According to the Appendix, values of the bifurcation loads for the FSDT (A62) and the S-FSDT (A54) are identical and slightly lower by the factor 1/(1 + η) than the CPT. For the data assumed in the analysis, according to (A63), we have η = 0.000564, which corresponds to 1/(1 + η) = 0.9994. As can be seen, according to (A63), corrections for the S-FSDT and the FSDT are very inconsiderable when compared to the CPT for the assumed ratio of (*h/a* = 0.01). The results obtained within the three theories are in conformity with the FEM outcomes.

The determined value of the critical stress (A43) for the CPT was introduced into the relationship for the total equivalent Kirchhoff force ${\hat{Q}}_{x}^{C}$ (A47a) and then the component ${\hat{Q}}_{x}^{C}$ when $p=p_{cr}^{C}$, is equal to

(1) $${\hat{Q}}_{x}^{C}\left(p=p_{cr}^{C}\right)=\left[\left(3-\nu \right)D{\left(\frac{\pi }{a}\right)}^{3}W-p_{cr}^{C}hW\frac{\pi }{a}\right]cos\frac{\pi x}{a}sin\frac{\pi y}{a}\;-\frac{EhW^{3}}{8}{\left(\frac{\pi }{a}\right)}^{3}cos\frac{\pi x}{a}sin\frac{\pi y}{a}cos\frac{2\pi y}{a}\;=-\left(1+\nu \right){\left(\frac{\pi }{a}\right)}^{3}DWcos\frac{\pi x}{a}sin\frac{\pi y}{a}-\frac{EhW^{3}}{8}{\left(\frac{\pi }{a}\right)}^{3}cos\frac{\pi x}{a}sin\frac{\pi y}{a}cos\frac{2\pi y}{a}$$

As can be easily noticed, in (1) there is a minus sign at both terms of the right-hand side. However, mutual relations depend on the relationships of products of trigonometric functions. The first term ${\hat{Q}}_{x}^{C}\left(p=p_{cr}^{C}\right)$ in (1) attains extreme values for *x =* 0; *a* and *y = a/*2 and the second respectively minimum for *x =* 0; *a* and *y = a/*2. Attention should be drawn to the fact that when *x =* 0 and *y = a/*2, the first term has a minus sign, and the second term has a plus sign. The opposite situation takes place when *x = a* and *y = a/*2, i.e., a plus sign is in the first term and a minus sign is in the second. The extreme values ${\hat{Q}}_{x}^{C}$ are attained inside the square plate.

A further analysis dealt with postbuckling states. According to (A39c), the force component *N*_xy_ equals zero, and one of two membrane components of transverse forces (according to (A12), (A23) and (A32)) vanishes as well.

Next, maximal absolute values of components of transverse forces in bending, membrane components (or in compression) or total forces for the three theories, determined according to the formulas given in Appendix A.2 for five overload values of critical load, i.e., $1.2\leq p^{\theta }/p_{cr}^{\theta }\leq 2.0$ (where the index θ=C,S,F), are listed in Table 2. In this table, values of the dimensionless deflection *W/h* and 1/(1 + η) are also presented.

For the CPT, the equivalent Kirchhoff forces $Q_{x}^{C},Q_{y}^{C}$ are equal according to (A45). On the other hand, values of membrane components of the transverse forces ${\bar{Q}}_{x}^{C},{\bar{Q}}_{y}^{C}$ differ depending on the overload $p^{\theta }/p_{cr}^{\theta }$. For the overload equal to 1.2, the ratio of maximal absolute components ${\bar{Q}}_{x}^{C}/{\bar{Q}}_{y}^{C}$ equals almost 5, whereas for the overload equal to 2, the ratio of membrane components is 1.4. The membrane forces ${\bar{Q}}_{x}^{C},{\bar{Q}}_{y}^{C}$ are independent of membrane deformation. It results from the fact that the membrane force $Q_{x}^{C}$ has a term linearly dependent on deflection and in the third power, which for $Q_{y}^{C}$ is in the third power only. A detailed analysis can be found in Appendix A.2. Components of the total equivalent Kirchhoff force ${\hat{Q}}_{y}^{C}$ are always higher for the range of loads under analysis than ${\hat{Q}}_{x}^{C}$. It follows from the term that is linear with respect to *W*, dependent on the overload ${\hat{Q}}_{x}^{C}$.

**Figure 2:**
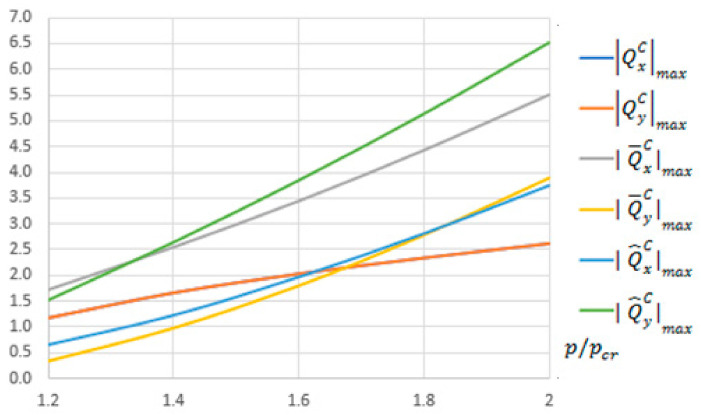
Maximal absolute values of components of the transverse forces ${\left|Q_{x}^{C}\right|}_{\max},{\left|Q_{y}^{C}\right|}_{\max},{\left|{\bar{Q}}_{x}^{C}\right|}_{\max},{\left|{\bar{Q}}_{y}^{C}\right|}_{\max},{\left|{\hat{Q}}_{x}^{C}\right|}_{\max},{\left|{\hat{Q}}_{y}^{C}\right|}_{\max}$ in N/mm for the CPT.

In Figure 2, the maximal absolute values of components of transverse Kirchhoff forces for the CPT versus $p^{C}/p_{cr}^{C}$, listed in Table 2, are presented.

For the S-FSDT, the maximal absolute values of components of the transverse forces ${\left|{\hat{Q}}_{x}^{S}\right|}_{max}$ and ${\left|{\hat{Q}}_{y}^{S}\right|}_{max}$ are the same in practice as for the CPT, which results from a very low value of the correction η. For the FSDT, force components in bending are 1.35 times lower for the CPT and the S-FSDT (cf. Formulas (A64) and (A45)). Similarly as for the S-FSDT, components of the total transverse force ${\hat{Q}}_{y}^{F}$ are always larger than ${\hat{Q}}_{x}^{F}$. When total transverse forces are accounted for in the CPT, the S-FSDT and the FSDT, they yield higher values than the equivalent Kirchhoff force by approx. 1.5 times for the component with respect to the *x*-axis (i.e., with a lower index *x*) and more than 2 times for the component with respect to the *y*-axis.

For the FEM, the values of components of the transverse forces ${\left|Q_{x}^{A}\right|}_{max}$ are higher than ${\left|Q_{y}^{A}\right|}_{max}$. At the overload equal to 1.2, the ratio ${\left|Q_{x}^{A}\right|}_{max}$/${\left|Q_{x}^{A}\right|}_{max}$ is 1.05, but for the overload of 2.0, it is equal to 1.36, respectively. The values ${\left|Q_{x}^{A}\right|}_{max}$ and ${\left|Q_{y}^{A}\right|}_{max}$ are closest to the equivalent Kirchhoff force $Q_{x}^{C},Q_{y}^{C}$. Thus, the transverse forces ${\left|Q_{x}^{A}\right|}_{max}$, ${\left|Q_{y}^{A}\right|}_{max}$ determined within the FEM have a different character than the total transverse forces for the CPT, the S-FSDT and the FSDT, determined on the basis of components in bending and compression. It can originate from the fact that membrane components were neglected in the FEM analysis.

**Figure 3:**
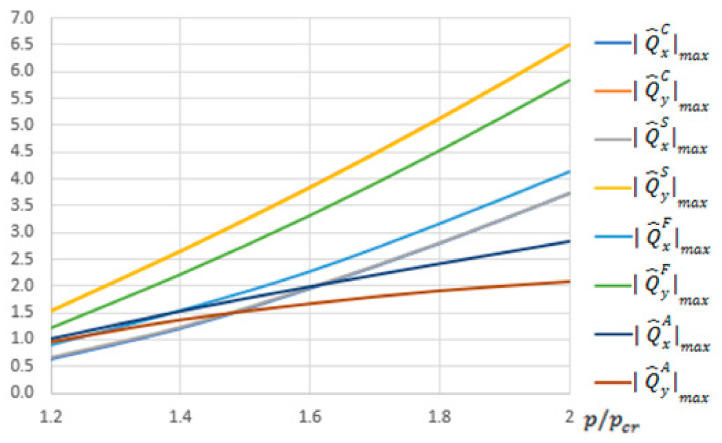
Maximal absolute values of the total transverse forces, ${\left|Q_{x}^{S}\right|}_{\max},{\left|Q_{y}^{S}\right|}_{\max},{\left|Q_{x}^{F}\right|}_{\max},{\left|Q_{y}^{F}\right|}_{\max},{\left|Q_{x}^{A}\right|}_{\max},{\left|Q_{y}^{A}\right|}_{\max}$ in N/mm for the CPT, the S-FSDT, the FSDT and the finite element method (FEM).

In Figure 3, the maximal absolute values of transverse resultant forces for the CPT, the S-FSDT and the FSDT (of which the values are listed in Table 2) versus overload are collected. The results for ANSYS are shown as well.

The present study was primarily aimed at drawing attention to a necessity to consider the effect of membrane components on total transverse forces for $1.2\leq p^{\theta }/p_{cr}^{\theta }\leq 2.0$.

**Figure 4:**
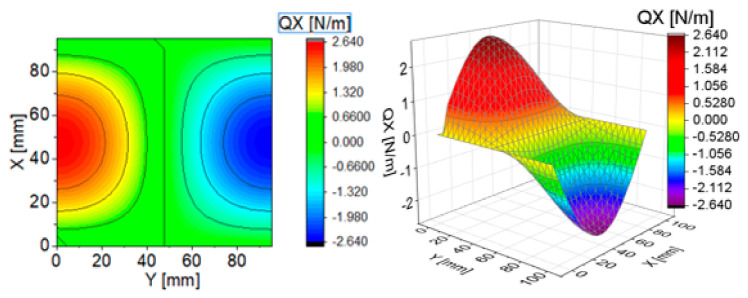
Contour-surface chart of $Q_{x}^{C}$

**Figure 5:**
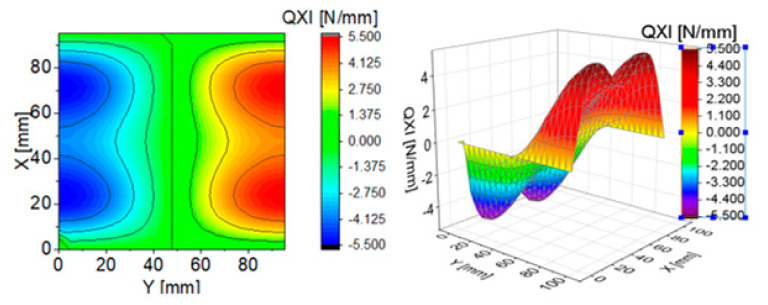
Contour-surface chart of ${\overset{¯}{Q}}_{x}^{C}$

**Figure 6:**
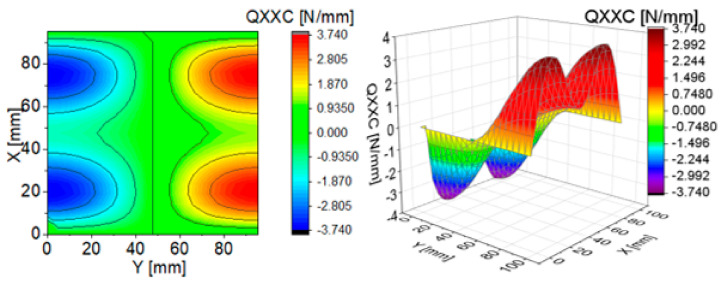
Contour-surface chart of ${\hat{Q}}_{x}^{C}$

**Figure 7:**
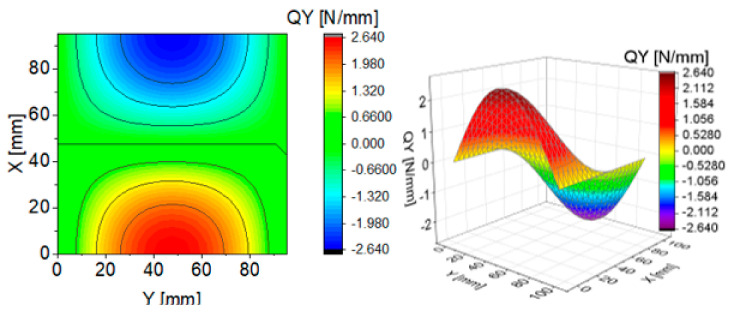
Contour-surface chart of $Q_{y}^{C}$

**Figure 8:**
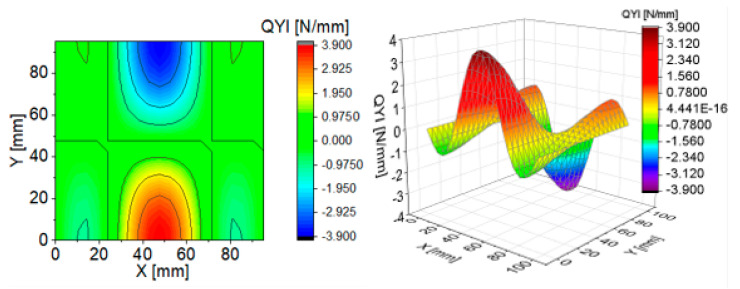
Contour-surface chart of ${\overset{¯}{Q}}_{y}^{C}$

**Figure 9:**
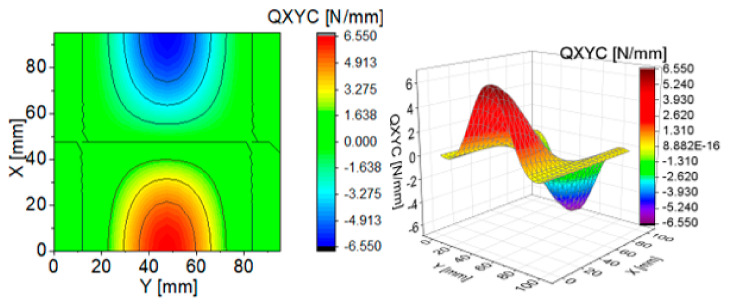
Contour-surface chart of ${\hat{Q}}_{y}^{C}$

To illustrate the effect of components of transverse Kirchhoff forces for the CPT, their distributions are presented in subsequent figures (Figure 4, Figure 5, Figure 6, Figure 7, Figure 8 and Figure 9). The components are shown in the contour drawings (denoted as a) and plane drawings (denoted as b) for the whole square plate and the overload $p^{C}/p_{cr}^{C}$ = 2 in the following sequence: $Q_{x}^{C}$ (Figure 4), ${\bar{Q}}_{x}^{C}$ (Figure 5), ${\hat{Q}}_{x}^{C}$ (Figure 6), $Q_{y}^{C}$ (Figure 7), ${\bar{Q}}_{y}^{C}$ (Figure 8) and ${\hat{Q}}_{y}^{C}$ (Figure 9).

The distributions of $Q_{x}^{C}$ and $Q_{y}^{C}$ presented in Figure 4 and Figure 7 are the same according to (A45). When the distributions of membrane forces are compared, the distribution for ${\bar{Q}}_{x}^{C}$ (Figure 5) is more complex than ${\bar{Q}}_{y}^{C}$ (Figure 8). However, in the authors’ opinion, the total transverse forces ${\hat{Q}}_{x}^{C}$ (Figure 6) and ${\hat{Q}}_{y}^{C}$ (Figure 9), which should be employed in failure criteria, are the most important. The components ${\hat{Q}}_{y}^{C}$ are larger than ${\hat{Q}}_{x}^{C}$, opposite to what happens in the FEM (Table 2).

It should be underlined once again that for the square plate under analysis, one of two membrane force components, which is dependent nonlinearly on the deflection *W*, equals zero (i.e., for *N*_xy_ = 0).

## 4. Conclusions

The effect of membrane components of transverse forces on total transverse forces within the three theories: the CPT, the S-FSDT and the FSDT, was discussed. When membrane components are taken into consideration, an increase can be observed in transverse forces equal to 1.5 times, at least for the square plate, freely supported along the whole circumference under consideration. It results from the fact that membrane components of transverse forces depend nonlinearly on the plate deflection. The results were compared to the FEM. The membrane transverse forces are independent of membrane deformation.

In composite materials, a failure of the structure resulting from delamination exerts a considerable effect on its integrity and load-carrying capacity. Transverse shear effects significantly influence the behaviour of composites. In the composite failure criteria, the impact of transverse force components in compression is neglected. From the authors’ viewpoint, these components prevail in the postbuckling state, which was proven in this study and should be considered in the failure criteria of composites, for instance, the Hashin failure criterion for 3D, LaRC04(3D), Matrix Failure under the additional condition that $\sigma_{33}=0$.

## Figures and Tables

**Table 1:** Values of critical stresses according to the classical plate theory (CPT), the simple first-order shear deformation theory (S-FSDT), the first-order shear deformation theory (FSDT) and ANSYS.

Critical Stresses in MPa			
CPT	S-FSDT	FSDT	ANSYS (FEM)
$p_{cr}^{C}$	$p_{cr}^{S}$	$p_{cr}^{F}$	$p_{cr}^{A}$
72.30	72.26	72.26	72.30

**Table 2:** Values of components of transverse forces for the CPT, the S-FSDT, the FSDT and ANSYS.

Theory	Symbol	Unit	$p^{\theta }/p_{cr}^{\theta }$ (Where the Index θ=C,S,F,A)				
			1.2	1.4	1.6	1.8	2.0
CPT (index C)	W/h	-	0.7656	1.083	1.326	1.531	1.712
	$1/\left(1+\eta \right)$	-	1.0				
	${\left|Q_{x}^{C}\right|}_{max}$	N/mm	1.17	1.66	2.03	2.34	2.62
	${\left|Q_{y}^{C}\right|}_{max}$	N/mm	1.17	1.66	2.03	2.34	2.62
	${\left|{\bar{Q}}_{x}^{C}\right|}_{max}$	N/mm	1.73	2.55	3.44	4.42	5.49
	${\left|{\bar{Q}}_{y}^{C}\right|}_{max}$	N/mm	0.347	0.983	1.80	2.78	3.88
	${\left|{\hat{Q}}_{x}^{C}\right|}_{max}$	N/mm	0.651	1.22	1.96	2.80	3.73
	${\left|{\hat{Q}}_{y}^{C}\right|}_{max}$	N/mm	1.52	2.64	3.84	5.13	6.51
S-FSDT (index S)	W/h	-	0.7651	1.0823	1.325	1.530	1.711
	$1/\left(1+\eta \right)$	-	0.9994				
	${\left|{\hat{Q}}_{x}^{S}\right|}_{max}$	N/mm	0.652	1.22	1.96	2.80	3.73
	${\left|{\hat{Q}}_{y}^{S}\right|}_{max}$	N/mm	1.52	2.64	3.83	5.12	6.51
FSDT (index F)	W/h	-	0.7651	1.0823	1.325	1.530	1.711
	$1/\left(1+\eta \right)$	-	0.9994				
	${\left|{\hat{Q}}_{x}^{F}\right|}_{max}$	N/mm	0.898	1.54	2.27	3.16	4.13
	${\left|{\hat{Q}}_{y}^{F}\right|}_{max}$	N/mm	1.21	2.21	3.31	4.52	5.83
FEM (index A)	W/h	-	0.76	1.07	1.31	1.50	1.68
	${\left|Q_{x}^{A}\right|}_{max}$	N/mm	1.00	1.52	1.98	2.41	2.83
	${\left|Q_{y}^{A}\right|}_{max}$	N/mm	0.96	1.37	1.67	1.91	2.08

**Figure A1:**
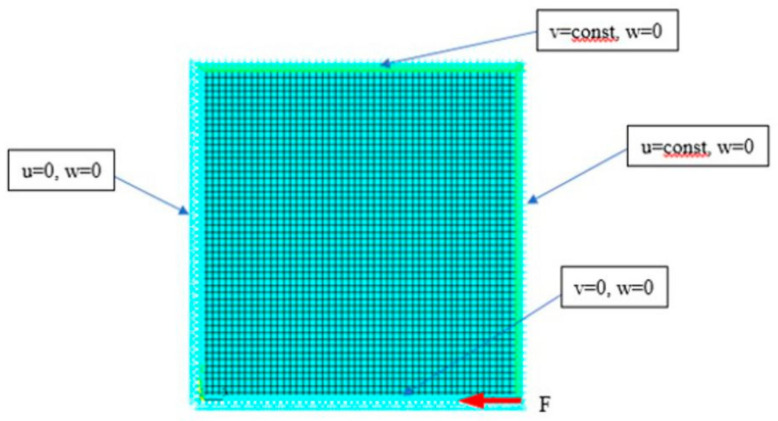
FEM model of the plate.

## Appendix A

### Appendix A.1. Governing Equations in the FSDT, the S-FSDT and the CPT

In this Appendix A, governing equations for three theories, namely the first-order shear deformation plate theory (FSDT), the simple first-order shear deformation theory (S-FSDT) and the classical plate theory (CPT), are presented. The equilibrium equations and the boundary conditions are attained within a variational approach.

The following geometrical relationships for the plate component are assumed [36,44,46]

(A1a) $$\varepsilon_{x}=u_{,x}+\frac{1}{2}w_{,x}^{2}$$

(A1b) $$\varepsilon_{y}=v_{,y}+\frac{1}{2}w_{,y}^{2}$$

(A1c) $$2\varepsilon_{xy}=\gamma_{xy}=u_{,y}+v_{,x}+w_{,x}w_{,y}$$

And

(A2) $$\kappa_{x}=-\psi_{x,x}\;\kappa_{y}=-\psi_{y,y}\;\kappa_{xy}=-\left(\psi_{x,y}+\psi_{y,x}\right)$$

where u, v are components of the plate displacement vector along the axis x, y, correspondingly, w is the total displacement vector along the *z*-axis and $\psi_{x}$, $\psi_{y}$ are the rotation angles of a transverse normal due to bending of the axes x, y, respectively, whereas the plane x−y overlaps the central plane before its buckling.

In this approach to the plate theory when transverse shear is accounted for, it is assumed that the total rotation angles of the normal to the central plane in two planes are, respectively [19,44]

(A3) $$w_{,x}=\psi_{x}+\beta_{x}\;\;\;w_{,y}=\psi_{y}+\beta_{y}$$

where $\beta_{x},\;\beta_{y}$ are the transverse shear angles.

Internal cross-sectional forces can be expressed in the form [19,44]

(A4a) $$N_{x}=\frac{Eh}{1-\nu^{2}}\left(\varepsilon_{x}+\nu \varepsilon_{y}\right)$$

(A4b) $$N_{y}=\frac{Eh}{1-\nu^{2}}\left(\varepsilon_{y}+\nu \varepsilon_{x}\right)$$

(A4c) $$N_{xy}=\frac{Eh}{1-\nu^{2}}\frac{1-\nu }{2}\gamma_{xy}$$

(A5a) $$M_{x}=-D\left(\psi_{x,x}+\nu \psi_{y,y}\right)$$

(A5b) $$M_{y}=-D\left(\psi_{y,y}+\nu \psi_{x,x}\right)$$

(A5c) $$M_{xy}=-D\frac{1-\nu }{2}\left(\psi_{x,y}+\psi_{y,x}\right)$$

(A6a) $$Q_{x}=k^{2}Gh\left(w_{,x}-\psi_{x}\right)$$

(A6b) $$Q_{y}=k^{2}Gh\left(w_{,y}-\psi_{y}\right)$$

The coefficient $k^{2}$ occurring in Formulas (A6) is known as the Mindlin correction factor.

The total potential energy Π of a thin rectangular plate of the following dimensions: ℓ×b×h can be written as [36]

(A7) $$\Pi =U-W=\frac{1}{2}\int_{0}^{\ell }\int_{0}^{b}\left(N_{x}\varepsilon_{x}+N_{y}\varepsilon_{y}+N_{xy}\gamma_{xy}\right)dxdy\;-\frac{1}{2}\int_{0}^{\ell }\int_{0}^{b}\left[M_{x}\psi_{x,x}+M_{y}\psi_{y,y}+M_{xy}\left(\psi_{x,y}+\psi_{y,x}\right)\right]dxdy\;+\frac{1}{2}\int_{0}^{\ell }\int_{0}^{b}[Q_{x}\left(w_{,x}-\psi_{x}\right)+Q_{y}\left(w_{,y}-\psi_{y}\right)]dxdy-\int_{0}^{b}hp^{0}\left(y\right)u\left|{}_{x=0}^{x=\ell }\right.dy$$

where *U* is the elastic strain internal energy, *W* is the work of external forces and *p^0^*(*y*) is the plate prebuckling external load in the central plane along the *x*-axis.

#### Appendix A.1.1. FSDT

To determine a variation of the total energy Π (A7), the following relations were substituted: (A1), (A2) and (A4)–(A6). Having grouped the terms including the same variations and summed each group of the terms to zero (due to mutual independence of the variations), the following equations are obtained

- Equations of equilibrium:
  (A8a) $$\int_{0}^{\ell }\int_{0}^{b}\left[N_{x,x}+N_{xy,y}\right]\delta udxdy=0$$
  (A8b) $$\int_{0}^{\ell }\int_{0}^{b}\left[N_{xy,x}+N_{y,y}\right]\delta vdxdy=0$$
  (A8c) $$\int_{0}^{\ell }\int_{0}^{b}[Q_{x,x}+Q_{y,y}+{\left(N_{x}w_{,x}+N_{xy}w_{,y}\right)}_{,x}+\left(N_{xy}w_{,x}+N_{y}w_{,y}{)}_{,y}\right]\delta wdxdy=0$$
  (A8d) $$\int_{0}^{\ell }\int_{0}^{b}\left[M_{x,x}+M_{xy,y}-Q_{x}\right]\delta \psi_{x}dxdy=0$$
  (A8e) $$\int_{0}^{\ell }\int_{0}^{b}\left[M_{xy,x}+M_{y,y}-Q_{y}\right]\delta \psi_{y}dxdy=0$$
- Boundary conditions
  for *x* = *const*
  (A9a) $$\int_{0}^{b}{\left[N_{x}-hp^{0}\left(y\right)\right]}_{}\delta udy\left|x=const=0\right.$$
  (A9b) $$\int_{0}^{b}N_{xy}{}_{}\delta vdy\left|x=const=0\right.$$
  (A9c) $$\int_{0}^{b}\left[N_{xy}w_{,y}+N_{x}w_{,x}+Q_{x}\right]\delta wdy\left|x=const\right.=0$$
  (A9d) $$\int_{0}^{b}\left[M_{x}\right]\delta \psi_{x}dy\left|x=const\right.=0$$
  (A9e) $$\int_{0}^{b}\left[M_{xy}\right]\delta \psi_{y}dy\left|x=const\right.=0$$
  for *y* = *const*
  (A10a) $$\int_{0}^{\ell }N_{y}{}_{}\delta vdx\left|y=const=0\right.$$
  (A10b) $$\int_{0}^{\ell }N_{xy}{}_{}\delta udx\left|y=const=0\right.$$
  (A10c) $$\int_{0}^{\ell }\left[N_{xy}w_{,x}+N_{y}w_{,y}+Q_{y}\right]\delta wdx\left|y=const\right.=0$$
  (A10d) $$\int_{0}^{\ell }\left[M_{xy}\right]\delta \psi_{x}dx\left|y=const\right.=0$$
  (A10e) $$\int_{0}^{\ell }\left[M_{y}\right]\delta \psi_{y}dx\left|y=const\right.=0$$

Equation (A8) is a system of equilibrium equations and relationships, and (A9) and (A10) are boundary conditions for *x = const* and *y = const*, respectively. Equations (A8)–(A10) hold for the FSDT. The above equations were determined from variational methods for which, according to Stokes’ theorem, the surface integral can be transformed into a circumference-oriented integral. The form the equations are presented follows from it.

According to (A6) and (A8d,e), transverse forces are expressed with the relations

(A11a) $$Q_{x}^{F}=M_{x,x}+M_{xy,y}$$

(A11b) $$Q_{y}^{F}=M_{y,y}+M_{xy,x}$$

where the upper index F was introduced for the FSDT. These are components of transverse forces dependent on derivatives of internal moments.

In Conditions (A9c) and (A10c) for the components of transverse forces, forces of the same characters are added, i.e., additional components of transverse forces that depend on projections of membrane forces, that is to say [36,45]

(A12a) $${\bar{Q}}_{x}^{F}=N_{x}w_{,x}+N_{xy}w_{,y}$$

(A12b) $${\bar{Q}}_{y}^{F}=N_{y}w_{,y}+N_{xy}w_{,x}$$

These forces do not affect membrane deformations.

When (A11) and (A12) are considered, according to (A9c) and (A10c), the total transverse forces ${\hat{Q}}_{x}^{F}$ and ${\hat{Q}}_{y}^{F}$ are introduced

(A13a) $${\hat{Q}}_{x}^{F}=Q_{x}^{F}+{\bar{Q}}_{x}^{F}=M_{x,x}+M_{xy,y}+N_{x}w_{,x}+N_{xy}w_{,y}$$

(A13b) $${\hat{Q}}_{y}^{F}=Q_{y}^{F}+{\bar{Q}}_{y}^{F}=M_{y,y}+M_{xy,x}+N_{y}w_{,y}+N_{xy}w_{,x}$$

#### Appendix A.1.2. S-FSDT

Next, the independent functions $\psi_{x},\;\psi_{y}$ (A2) were substituted with a potential function $\phi \left(x,y\right)$ [19,32,33,34,44] such that

(A14) $$\phi_{,x}=\psi_{x}\;\;\;\phi_{,y}=\psi_{y}$$

This approach consists of the fact that the function ϕ is treated as deflections in bending. Because the function ϕ is introduced, the Reissner boundary conditions [32], not considered in the present study, are ignored.

If $\beta_{x}=w_{s,x}$, $\beta_{y}=w_{s,y}$ hold for (A3), the shear deflection is obtained from the relationship $w_{s}=w-\phi$. From (A3) and (A14), it follows that

(A15) $$w_{,x}=\phi_{,x}+\beta_{x}=\phi_{,x}+w_{s,x}\;\;\;w_{,y}=\phi_{,y}+\beta_{y}=\phi_{,y}+w_{s,y}$$

When (A15) is accounted for, the internal forces (A5) and (A6) are written as

(A16a) $$M_{x}\;=\;-D\;(\phi_{x,x}\;+\;v\phi_{y,y})$$

(A16b) $$M_{y}\;=\;-D\;(\phi_{y,y}\;+\;v\phi_{x,x})$$

(A16c) $$M_{x,y}\;=\;-D\;(1\;-\;v)\;\phi_{x,y}$$

(A17a) $$Q_{x}=k^{2}Gh\left(w_{,x}-\phi_{,x}\right)$$

(A17b) $$Q_{y}=k^{2}Gh\left(w_{,y}-\phi_{,y}\right)$$

From the variation of the total energy Π (A7) with respect to the displacement variable components u, v, w, ϕ, we obtain

- Equations of equilibrium
  (A18a) $$\int_{0}^{\ell }\int_{0}^{b}\left[N_{x,x}+N_{xy,y}\right]\delta udxdy=0$$
  (A18b) $$\int_{0}^{\ell }\int_{0}^{b}\left[N_{xy,x}+N_{y,y}\right]\delta vdxdy=0$$
  (A18c) $$\int_{0}^{\ell }\int_{0}^{b}[Q_{x,x}+Q_{y,y}+{\left(N_{x}w_{,x}+N_{xy}w_{,y}\right)}_{,x}+\left(N_{xy}w_{,x}+N_{y}w_{,y}{)}_{,y}\right]\delta wdxdy=0$$
  (A18d) $$\int_{0}^{\ell }\int_{0}^{b}\left[M_{x,xx}+2M_{xy,xy}+M_{y,yy}-Q_{x,x}-Q_{y,y}\right]\delta \phi dxdy=0$$
- Boundary conditions
  for *x* = *const*
  (A19a) $$\int_{0}^{b}{\left[N_{x}-hp^{0}\left(y\right)\right]}_{}\delta udy\left|x=const=0\right.$$
  (A19b) $$\int_{0}^{b}N_{xy}{}_{}\delta vdy\left|x=const=0\right.$$
  (A19c) $$\int_{0}^{\ell }\left[N_{xy}w_{,y}+N_{x}w_{,x}+Q_{x}\right]\delta wdy\left|x=const\right.=0$$
  (A19d) $$\int_{0}^{b}\left[2M_{xy,y}+M_{x,x}-Q_{x}\right]\delta \phi dy\left|x=const=0\right.$$
  (A19e) $$\int_{0}^{b}\left[M_{x}\right]\delta \phi_{,x}dy\left|x=const=0\right.$$
  for *y* = *const*
  (A20a) $$\int_{0}^{\ell }N_{y}{}_{}\delta vdx\left|y=const=0\right.$$
  (A20b) $$\int_{0}^{\ell }N_{xy}{}_{}\delta udx\left|y=const=0\right.$$
  (A20c) $$\int_{0}^{\ell }\left[N_{xy}w_{,x}+N_{y}w_{,y}+Q_{y}\right]\delta wdx\left|y=const\right.=0$$
  (A20d) $$\int_{0}^{\ell }\left[2M_{xy,x}+M_{y,y}-Q_{y}\right]\delta \phi dx\left|y=const=0\right.$$
  (A20e) $$\int_{0}^{\ell }\left[M_{y}\right]\delta \phi_{,y}dx\left|y=const=0\right.$$
  for the plate corner, i.e., for *x* = *const* and *y = const*
  (A21) $$2M_{xy}\delta \phi \left|x=const\right.\left|y=const=0\right.$$

Equations (A18)–(A21) correspond to the S-FSDT, i.e., to a two-variable refined plate theory.

According to (A6), (A19d) and (A20d), the transverse forces dependent on the variable ϕ are expressed as

(A22a) $$Q_{x}^{S}=M_{x,x}+2M_{xy,y}$$

(A22b) $$Q_{y}^{S}=M_{y,y}+2M_{xy,x}$$

where the upper index S was introduced for the S-FSDT.

The membrane components of the transverse forces dependent on the variable w, according to (A19c) and (A20c), take the form

(A23a) $${\bar{Q}}_{x}^{S}=N_{x}w_{,x}+N_{xy}w_{,y}$$

(A23b) $${\bar{Q}}_{y}^{S}=N_{y}w_{,y}+N_{xy}w_{,x}$$

Analogously to (A13), the total transverse forces ${\hat{Q}}_{x}^{S}$ and ${\hat{Q}}_{y}^{S}$ for the S-FSDT are as follows

(A24a) $${\hat{Q}}_{x}^{S}=Q_{x}^{S}+{\bar{Q}}_{x}^{S}=M_{x,x}+2M_{xy,y}+N_{x}w_{,x}+N_{xy}w_{,y}$$

(A24b) $${\hat{Q}}_{y}^{S}=Q_{y}^{S}+{\bar{Q}}_{y}^{S}=M_{y,y}+2M_{xy,x}+N_{y}w_{,y}+N_{xy}w_{,x}$$

Comparing the formulas for transverse forces in pairs of (A11) and (A22), as well as (A13) and (A24), it can be easily noticed that in the case of the S-FSDT, we have a coefficient two at a derivative of the torque $M_{xy}$, which for the FSDT is equal to one.

#### Appendix A.1.3. CPT

In the classical theory of plates (CPT), transverse forces are neglected (A6) and, moreover, in (A2) and (A3), it should be [36]

(A25) $$w_{,x}=\psi_{x}\;\;\;w_{,y}=\psi_{y}$$

Taking into consideration (A25) in (A5), we have [46]

(A26a) $$M_{x}=-D\left(w_{,xx}+\nu w_{,yy}\right)$$

(A26b) $$M_{y}=-D\left(w_{,yy}+\nu w_{,xx}\right)$$

(A26c) $$M_{xy}=-D\left(1-\nu \right)w_{,xy}$$

When the above-mentioned relations are accounted for and it is assumed that $Q_{x}=Q_{y}=0$ in (A7), the following equations are obtained [46]

- Equations of equilibrium
  (A27a) $$\int_{0}^{\ell }\int_{0}^{b}\left[N_{x,x}+N_{xy,y}\right]\delta udxdy=0$$
  (A27b) $$\int_{0}^{\ell }\int_{0}^{b}\left[N_{xy,x}+N_{y,y}\right]\delta vdxdy=0$$
- Boundary conditions
  for *x* = *const*
  (A28a) $$\int_{0}^{b}{\left[N_{x}-hp^{0}\left(y\right)\right]}_{}\delta udy\left|x=const=0\right.$$
  (A28b) $$\int_{0}^{b}N_{xy}{}_{}\delta vdy\left|x=const=0\right.$$
  (A28c) $$\int_{0}^{\ell }\left[M_{x,x}+2M_{xy,y}+N_{xy}w_{,y}+N_{x}w_{,x}\right]\delta wdy\left|x=const\right.=0$$
  (A28d) $$\int_{0}^{b}M_{x}\delta w_{,x}dy\left|x=const=0\right.$$
  for *y* = *const*
  (A29a) $$\int_{0}^{\ell }N_{y}{}_{}\delta vdx\left|y=const=0\right.$$
  (A29b) $$\int_{0}^{\ell }N_{xy}{}_{}\delta udx\left|y=const=0\right.$$
  (A29c) $$\int_{0}^{\ell }\left[M_{y,y}+2M_{xy,x}+N_{xy}w_{,x}+N_{y}w_{,y}\right]\delta wdx\left|y=const\right.=0$$
  (A29d) $$\int_{0}^{\ell }\left[M_{y}\right]\delta w_{,y}dx\left|y=const=0\right.$$
  for the plate corner, i.e., for *x* = *const* and *y = const*
  (A30) $$2M_{xy}\delta w\left|x=const\right.\left|y=const=0\right.$$

In the history of the CPT, for the first two components in (A28c) and (A29c), a term of equivalent Kirchhoff transverse forces, was introduced and defined as

(A31a) $$Q_{x}^{C}=M_{x,x}+2M_{xy,y}$$

(A31b) $$Q_{y}^{C}=M_{y,y}+2M_{xy,x}$$

where the upper index C was used for the CPT.

By analogy to the FSDT and the S-FSDT, according to (A28c) and (A29c), the following components were assumed

(A32a) $${\bar{Q}}_{x}^{C}=N_{x}w_{,x}+N_{xy}w_{,y}$$

(A32b) $${\bar{Q}}_{y}^{C}=N_{y}w_{,y}+N_{xy}w_{,x}$$

The above-mentioned components of transverse forces result from the projection of membrane forces. Thus, they can be referred to as equivalent Kirchhoff membrane forces.

Taking into account the two above-mentioned systems, the total equivalent Kirchhoff transverse forces ${\hat{Q}}_{x}^{C}$ and ${\hat{Q}}_{y}^{C}$ for the CPT were written as [36,45,46]

(A33a) $${\hat{Q}}_{x}^{C}=Q_{x}^{C}+{\bar{Q}}_{x}^{C}=M_{x,x}+2M_{xy,y}+N_{x}w_{,x}+N_{xy}w_{,y}$$

(A33b) $${\hat{Q}}_{y}^{C}=Q_{y}^{C}+{\bar{Q}}_{y}^{C}=M_{y,y}+2M_{xy,x}+N_{y}w_{,y}+N_{xy}w_{,x}$$

Formulas (A24) and (A33) have the same structure. It should be remembered that for the S-FDST, these equations are for the two variables w,ϕ, whereas for the CPT, only for the variable w.

#### Appendix A.1.4. Shear Forces

When we compare relationships (A13) for the FSDT, (A24) for the S-FDST and (A32) for the CPT, we can see that the expressions for total transverse forces are identical in practice. In the FSDT, at the derivative of the torque *M_xy_* there is a coefficient equal to one and not two, as it takes place for the S-FSDT and the CPT. It is caused by additional boundary conditions (A9c) and (A10c) for the FSDT.

For postbuckling states, there are membrane forces in the central plane that yield simultaneously projections for additional components of transverse forces (A12), (A23) and (A32) for the FSDT, the S-FSDT and the CPT, correspondingly. In the case of postbuckling equilibrium paths, these additional components are larger than the components of transverse forces (A11), (A22) and (A31).

### Appendix A.2. Solution to Governing Equations within the FSDT, the S-FSDT and the CPT

A square isotropic plate freely supported along all edges, compressed along the *x*-axis (Figure 1), was analysed. The plate of the dimensions *a* and the thickness *h* was assumed to have the following material constants: Young’s modulus E and Poisson’s ratio ν. The considerations were limited to an elastic range.

#### Appendix A.2.1. Continuity Equation of Deformations (the So-Called Inseparability Equation of Deformations)

On the assumption that relations (A1) and (A4) hold for the three theories under consideration (i.e., the FSDT, the S-FSDT and the CPT), two first equations of equilibrium for each theory are the same (cf. (A8a,b), (A18a,b) and (A27a,b)). To solve them, a function of Airy forces *F* was introduced. It is defined as follows [36,45]

(A34a) $$N_{x}=\sigma_{x}h=F_{,yy}$$

(A34b) $$N_{y}=\sigma_{y}h=F_{,xx}$$

(A34c) $$N_{xy}=\tau_{xy}h=-F_{,xy}$$

Taking into consideration (A34) in (A8a,b), (A18a,b) and (A26a,b), it was found that both the equations were identically zero. That system of equations was substituted by one equation referred to as a continuity equation of deformations or an inseparability equation of deformations.

For this purpose, a system of Equations (A1) was rewritten as

(A35) $$\varepsilon_{x,yy}+\varepsilon_{y,xx}-\gamma_{xy,xy}=w_{,xy}^{2}-w_{,xx}w_{,xy}$$

When we take account of relations (A1), (A4), (A34) in the above-mentioned system, we obtain a continuity equation of deformations in the form [36,45]

(A36) $$\nabla \nabla F\equiv F_{,xxxx}+2F_{,xxyy}+F_{,yyyy}=E\left(w_{,xy}^{2}-w_{,xx}w_{,xy}\right)$$

The equation is linear with respect to *F* and nonlinear with respect to *w*.

The deflection of a square plate freely supported is approximated in the following way [36]

(A37) $$w=Wsin\frac{\pi x}{a}sin\frac{\pi y}{a}$$

which satisfies the following boundary conditions

(A38) $$w\left(x=0\right)=w\left(x=a\right)=w\left(y=0\right)=w\left(y=a\right)=0$$

When (A37) is substituted into (A36), a function of the membrane forces F is determined, and then components of the membrane forces are defined as [36]

(A39a) $$N_{x}=F_{,yy}=-\frac{EhW^{2}}{8}{\left(\frac{\pi }{a}\right)}^{2}cos\frac{2\pi y}{a}-ph$$

(A39b) $$N_{y}=F_{,xx}=-\frac{EhW^{2}}{8}{\left(\frac{\pi }{a}\right)}^{2}cos\frac{2\pi x}{a}$$

(A39c) $$N_{xy}=-F_{,xy}=0$$

where *p* is the stress along the *x*-axis (Figure 1).

The functions of forces (A39) fulfil the following boundary conditions [36,46]

(A40a) $$u\left(x=0\right)=u\left(x=a\right)=const\;\;N_{xy}\left(x=0\right)=N_{xy}\left(x=a\right)=0$$

(A40b) $$v\left(y=0\right)=v\left(y=a\right)=const\;\;N_{xy}\left(y=0\right)=N_{xy}\left(y=a\right)=0$$

#### Appendix A.2.2. Solution to the Nonlinear Problem of Stability for the CPT

Within the CPT, a solution to the nonlinear problem after an introduction of the force function (A34) is composed of two nonlinear Equations (A36) and (A27c) with respect to *F* and *w*, correspondingly. One of the solutions is Relationship (A39). The second solution is the equation of Equilibrium (A27c) with respect to *w*, which was solved with the Galerkin–Bubnov method [36].

The assumed function of deflection in (A37), according to (A26), fulfils the boundary conditions of free support

(A41) $$M_{x}\left(x=0\right)=M_{x}\left(x=a\right)=M_{y}\left(y=0\right)=M_{y}\left(y=a\right)=0$$

To solve the problem, Relationships (A37) and (A39) are introduced into Equation (A27c). Finally, we obtain an equation that describes the postbuckling equilibrium path for the CPT

(A42) $$p^{C}=p_{cr}^{C}+\frac{E\pi^{2}}{8a^{2}}W^{2}$$

where

(A43) $$p_{cr}^{C}=\frac{4D\pi^{2}}{ha^{2}}$$

Relationship (A42) can also be expressed as [36]

(A44) $$\left(1-\frac{p^{C}}{p_{cr}^{C}}\right)+0.34125{\left(\frac{W}{h}\right)}^{2}=0$$

Transverse forces in the CPT are referred to as equivalent Kirchhoff transverse forces (A31) and, according to (A37), expressed with the following relations

(A45a) $$Q_{x}^{C}=M_{x,x}+2M_{xy,y}=\left(3-\nu \right)D{\left(\frac{\pi }{a}\right)}^{3}Wcos\frac{\pi x}{a}sin\frac{\pi y}{a}$$

(A45b) $$Q_{y}^{C}=M_{y,y}+2M_{xy,x}=\left(3-\nu \right)D{\left(\frac{\pi }{a}\right)}^{3}Wsin\frac{\pi x}{a}cos\frac{\pi y}{a}$$

From (A32), for equivalent Kirchhoff forces in compression, taking account of (A3) and (A39), we have

(A46a) $${\bar{Q}}_{x}^{C}=N_{x}w_{,x}+N_{xy}w_{,y}=-\frac{EhW^{3}}{8}{\left(\frac{\pi }{a}\right)}^{3}cos\frac{\pi x}{a}sin\frac{\pi y}{a}cos\frac{2\pi y}{a}-phW\frac{\pi }{a}cos\frac{\pi x}{a}sin\frac{\pi y}{a}$$

(A46b) $${\bar{Q}}_{y}^{C}=N_{y}w_{,y}+N_{xy}w_{,x}=-\frac{EhW^{3}}{8}{\left(\frac{\pi }{a}\right)}^{3}sin\frac{\pi x}{a}cos\frac{\pi y}{a}cos\frac{2\pi x}{a}$$

For transversally inextensible membrane plates, the shear forces are independent of membrane deformation.

The relations for total equivalent Kirchhoff transverse forces result from the two above-mentioned systems of equations and (A33)

(A47a) $${\hat{Q}}_{x}^{C}=\left(3-\nu \right)D{\left(\frac{\pi }{a}\right)}^{3}Wcos\frac{\pi x}{a}sin\frac{\pi y}{a}-phW\frac{\pi }{a}cos\frac{\pi x}{a}sin\frac{\pi y}{a}\;-\frac{EhW^{3}}{8}{\left(\frac{\pi }{a}\right)}^{3}cos\frac{\pi x}{a}sin\frac{\pi y}{a}cos\frac{2\pi y}{a}$$

(A47b) $${\hat{Q}}_{y}^{C}=\left(3-\nu \right)D{\left(\frac{\pi }{a}\right)}^{3}Wsin\frac{\pi x}{a}cos\frac{\pi y}{a}-\frac{EhW^{3}}{8}{\left(\frac{\pi }{a}\right)}^{3}sin\frac{\pi x}{a}cos\frac{\pi y}{a}cos\frac{2\pi x}{a}$$

As one can easily notice, the first terms on right-hand sides in (A47) are positive, whereas the remaining terms are negative. The two first terms in (A47a) and the first term in (A47b) are a linear function of *W*, whereas the remaining terms are nonlinear with respect to *W*. To solve the problem, the deflection *W* should be determined from Equation (A44) for the given load *p*, and next the values of transverse forces (A45)–(A47).

The first component ${\hat{Q}}_{x}^{C}$ in (A47) depends formally on the value of the compressive load *p*, whereas the second ${\hat{Q}}_{y}^{C}$ does not. However, it should be remembered that for the given load $p>p_{cr}^{C}$, we have the deflection W, and thus, indirectly, the equivalent Kirchhoff transverse forces ${\hat{Q}}_{x}^{C}$ and ${\hat{Q}}_{y}^{C}$ depend on *p* and *W*.

#### Appendix A.2.3. Solution to the Nonlinear Problem of Stability for the S-FSDT

A solution to the nonlinear stability problem for the S-FSDT consists of three equations: (A36) and (A18c,d). The solutions to (A36) are relationships (A39). The solution to the remaining two equations, when we take into account (A37), is predicted in the form

(A48) $$\phi =\Phi sin\frac{\pi x}{a}sin\frac{\pi y}{a}$$

which, according to (A16) fulfils, the boundary conditions of free support

(A49) $$M_{x}\left(x=0\right)=M_{x}\left(x=a\right)=M_{y}\left(y=0\right)=M_{y}\left(y=a\right)=0$$

Having substituted (A37) and (A48) into (A18d), a linear relation between amplitudes of the functions *W* and Φ was determined as

(A50) $$W=\Phi \left(1+\eta \right)$$

where

(A51) $$\eta =\frac{2D}{k^{2}Gh}{\left(\frac{\pi }{a}\right)}^{2}$$

Nonlinear Equation (A18c) was solved within the Galerkin–Bubnov method, as for the CPT. For this purpose, Relations (A16), (A17), (A39) and (A50) were introduced into the above-mentioned equation, and after some transformations, an equation describing the postbuckling equilibrium path was obtained

(A52a) $$p^{S}=p_{cr}^{S}+\frac{EW^{2}}{8}{\left(\frac{\pi }{a}\right)}^{2}$$

where

(A52b) $$p_{cr}^{S}=\frac{4D\pi^{2}}{ha^{2}}\frac{1}{1+\eta }$$

Relation (A52) can be expressed in an analogous way to (A44) as

(A53) $$\left(1-\frac{p^{s}}{p_{cr}^{S}}\right)+0.34125\left(1+\eta \right){\left(\frac{W}{h}\right)}^{2}=0$$

From the formal point of view, Relationships (A42) and (A52) have an identical structure due load and the deflection W, and a difference lies in critical forces only. From a comparison of (A43) and (A53), we have

(A54) $$p_{cr}^{S}=\frac{4D\pi^{2}}{ha^{2}}\frac{1}{1+\eta }=p_{cr}^{C}\frac{1}{1+\eta }$$

The transverse components of forces in the S-FSDT, according to (A16), (A22) and (A50), are in the form

(A55a) $$Q_{x}^{S}=\left(3-\nu \right)D{\left(\frac{\pi }{a}\right)}^{3}\frac{W}{1+\eta }cos\frac{\pi x}{a}sin\frac{\pi y}{a}$$

(A55b) $$Q_{y}^{S}=\left(3-\nu \right)D{\left(\frac{\pi }{a}\right)}^{3}\frac{W}{1+\eta }sin\frac{\pi x}{a}cos\frac{\pi y}{a}$$

and, according to (A23), (A37), (A39) and (A48), we have

(A56a) $${\bar{Q}}_{x}^{S}=-\frac{EhW^{3}}{8}{\left(\frac{\pi }{a}\right)}^{3}cos\frac{\pi x}{a}sin\frac{\pi y}{a}cos\frac{2\pi y}{a}-phW\frac{\pi }{a}cos\frac{\pi x}{a}sin\frac{\pi y}{a}$$

(A56b) $${\bar{Q}}_{y}^{S}=-\frac{EhW^{3}}{8}{\left(\frac{\pi }{a}\right)}^{3}sin\frac{\pi x}{a}cos\frac{\pi y}{a}cos\frac{2\pi x}{a}$$

Systems of equations (A46) and (A56) have an identical structure.

From the above two systems of equations and (A24), we obtain

(A57a) $${\hat{Q}}_{x}^{S}=\left(3-\nu \right)D{\left(\frac{\pi }{a}\right)}^{3}\frac{W}{1+\eta }cos\frac{\pi x}{a}sin\frac{\pi y}{a}-phW\frac{\pi }{a}cos\frac{\pi x}{a}sin\frac{\pi y}{a}\;-\frac{EhW^{3}}{8}{\left(\frac{\pi }{a}\right)}^{3}cos\frac{\pi x}{a}sin\frac{\pi y}{a}cos\frac{2\pi y}{a}$$

(A57b) $${\bar{Q}}_{y}^{S}=\left(3-\nu \right)D{\left(\frac{\pi }{a}\right)}^{3}\frac{W}{1+\eta }sin\frac{\pi x}{a}cos\frac{\pi y}{a}-\frac{EhW^{3}}{8}{\left(\frac{\pi }{a}\right)}^{3}sin\frac{\pi x}{a}cos\frac{\pi y}{a}cos\frac{2\pi x}{a}$$

Comparing relations (A47) for the CPT and (A57) for the S-FSDT, it can be easily seen that the formulas differ only by the factor $1/\left(1+\eta \right)$ in the first term of right-hand sides of expressions in (A57). Moreover, the first two components of the right-hand side of (A57a) and the first term in (A57b) depend linearly on *W*, whereas the remaining ones are in the third power for *W*.

#### Appendix A.2.4. Solution to the Nonlinear Problem of Stability for the FSDT

For the FSDT, a solution to the nonlinear stability problem consists of a function of Airy forces *F* (A39) and a system of equilibrium equations (A8c–e). The solutions to these equations are the function *w* (A37) and the functions $\psi_{x},\psi_{y}$, which were assumed in the form

(A58a) $$\psi_{x}=\Psi_{x}cos\frac{\pi x}{a}sin\frac{\pi y}{a}$$

(A58b) $$\psi_{y}=\Psi_{y}sin\frac{\pi x}{a}cos\frac{\pi y}{a}$$

The function *w* (A37) fulfils the boundary conditions (A38), whereas the functions $\psi_{x},\psi_{y}$ satisfy the conditions of free support

(A59a) $$\psi_{x}\left(y=0\right)=\psi_{x}\left(y=a\right)=0$$

(A59b) $$\psi_{y}\left(x=0\right)=\psi_{y}\left(x=a\right)=0$$

(A59c) $$M_{x}\left(x=0\right)=M_{x}\left(x=a\right)=0$$

(A59d) $$M_{y}\left(y=0\right)=M_{y}\left(y=a\right)=0$$

After the substitution of (A58) into two equations (A8d,e), the following linear dependencies between the functions $W,\Psi_{x},\Psi_{y}$ are attained

(A60) $$\Psi_{x}=\Psi_{y}=\frac{W}{1+\eta }\left(\frac{\pi }{a}\right)$$

where relation (A51) holds.

A further step was a solution to the nonlinear Equation (A51), which describes the postbuckling equilibrium path. This solution was obtained employing, similarly as for the CPT and the S-FSDT, the Galerkin–Bubnov method. The postbuckling equation of equilibrium has the form

(A61a) $$p^{F}=p_{cr}^{F}+\frac{EW^{2}}{8}{\left(\frac{\pi }{a}\right)}^{2}$$

or

(A61b) $$\left(1-\frac{p^{F}}{p_{cr}^{F}}\right)+0.34125\left(1+\eta \right){\left(\frac{W}{h}\right)}^{2}=0$$

where

(A62) $$p_{cr}^{F}=\frac{4D\pi^{2}}{ha^{2}}\frac{1}{1+\eta }=p_{cr}^{S}=p_{cr}^{C}\frac{1}{1+\eta }$$

As can be easily seen in (A62), the values of critical loads for the S-FSDT and the FSDT are identical. The postbuckling equilibrium paths for the S-FSDT (A52), (A52a) and the FSDT (A61), (A61a) are the same, and, additionally, they differ from the CPT only by the factor $1/\left(1+\eta \right)$.

In a comparison of the Kirchhoff theory (i.e., CPT) with the Mindlin (the S-FSDT version) and Reissner (FSDT) theories, the reduction factor η plays a very important role (A51).

For the CPT, the factor η, according to [3,46], can be written for the case when G→∞, which leads to the relation η=0.

The reduction factor η (A51) can be transformed to

(A63) $$\eta =\frac{2D}{k^{2}Gh}{\left(\frac{\pi }{a}\right)}^{2}=\frac{\pi^{2}}{3k^{2}\left(1-\nu \right)}{\left(\frac{h}{a}\right)}^{2}$$

For $k^{2}=5/6$, ν=0.3 from (A63), we obtain $\eta =5.64_{}{\left(h/a\right)}^{2}$. When *h/a* = 0.1, η=0.0564; whereas when h/a=0.04, η=0.009. That means that for the plate of medium thickness (when h/a=0.1), the correction for the S-FSDT and the FSDT when compared to the CPT is lower than 6%. When h/a = 0.04, it is less than 1%. For thin plates (i.e., when h/a<0.05), the reduction factor η < 0.014.

In the last stage, the determined components of transverse forces in the FSDT, according to (A60), (A37) and (A8), were written as

(A64) $$Q_{x}^{F}=2D{\left(\frac{\pi }{a}\right)}^{3}\frac{W}{1+\eta }cos\frac{\pi x}{a}sin\frac{\pi y}{a}\;Q_{y}^{F}=2D{\left(\frac{\pi }{a}\right)}^{3}\frac{W}{1+\eta }sin\frac{\pi x}{a}cos\frac{\pi y}{a}$$

and additionally, according to (A9), (A10), (A39) and (A58), we have

(A65) $${\bar{Q}}_{x}^{F}=-\frac{EhW^{3}}{8}{\left(\frac{\pi }{a}\right)}^{3}cos\frac{\pi x}{a}sin\frac{\pi y}{a}cos\frac{2\pi y}{a}-phW\frac{\pi }{a}cos\frac{\pi x}{a}sin\frac{\pi y}{a}\;{\bar{Q}}_{y}^{F}=-\frac{EhW^{3}}{8}{\left(\frac{\pi }{a}\right)}^{3}sin\frac{\pi x}{a}cos\frac{\pi y}{a}cos\frac{2\pi x}{a}$$

Dependencies (A46), (A56) and (A65) have the same structure for the CPT, the S-FSDT and the FSDT. These are transverse force components dependent on membrane forces.

From the system of the above two equations and (A24), we obtain

(A66a) $${\hat{Q}}_{x}^{F}=2D{\left(\frac{\pi }{a}\right)}^{3}\frac{W}{1+\eta }cos\frac{\pi x}{a}sin\frac{\pi y}{a}-phW\frac{\pi }{a}cos\frac{\pi x}{a}sin\frac{\pi y}{a}\;-\frac{EhW^{3}}{8}{\left(\frac{\pi }{a}\right)}^{3}cos\frac{\pi x}{a}sin\frac{\pi y}{a}cos\frac{2\pi y}{a}$$

(A66b) $${\bar{Q}}_{y}^{F}=2D{\left(\frac{\pi }{a}\right)}^{3}\frac{W}{1+\eta }sin\frac{\pi x}{a}cos\frac{\pi y}{a}-\frac{EhW^{3}}{8}{\left(\frac{\pi }{a}\right)}^{3}sin\frac{\pi x}{a}cos\frac{\pi y}{a}cos\frac{2\pi x}{a}$$

When Relations (A47) for the CPT and (A57) for the S-FSDT are compared, one can easily see that they differ in the factor $1/\left(1+\eta \right)$ only in the first term of right-hand sides of expressions (A57).

Like for the CPT and the S-FSDT, the first two terms of the right-hand side (A66a) and the first term in (A66b) depend linearly on *W*, whereas the remaining terms are in the third power for *W*.

#### Appendix A.2.5. Solution to the Nonlinear Problem of Stability in the FEM (ANSYS)

In the given system of coordinates (Figure A1), a square plate jointly supported was investigated. The support was achieved taking the degrees of freedom away along the following directions: *x* for *x =* 0 (displacement *u =* 0), *y* for *y* = 0 (displacement *v =* 0), *z* for *z =* 0 (displacement *w =* 0, perpendicular to the central plane of the plate).

Numerical calculations were performed with the commercial ANSYS^®^ software [47] based on the FEM. In Figure A1, a compression force along the *x*-direction, applied to the node, is indicated. The displacements u and v for x = a and y = a (a-plate length/width), respectively, were assumed to be constant, using the *Couple Dof’s* function, which allows for controlling a group of nodes (the so-called *Slaves*) with one main node (the so-called *Master*). In the numerical model, a Shell181 finite element with six degrees of freedom was used. The FEM model had 2500 elements, 15,606 degrees of freedom, and the element size was 2 mm. In the nonlinear problem, an option of *Update geom*, i.e., a possibility to impose an initial shape imperfection, which is a solution to the plate postbuckling state, was involved. In the computations, the imperfection amplitude equal to *w0* = 0.0001 h was assumed. The problem was solved within the Newton–Raphson method for the system of equilibrium equations, which is a system of algebraic equations, for 1000 load steps within the range $1.001\leq P/P_{cr}\leq 2.000$.

In the Shell181 element description [46], the transverse forces are denoted as $Q_{13}$ and $Q_{23}$. For uniform notations in the presented study, it is assumed that

(A67) $$Q_{x}^{A}\equiv Q_{13}\;\;\;Q_{y}^{A}\equiv Q_{23}$$

where the index A refers to ANSYS^®^ [47] based on the FEM.

In FEM procedures, the manufacturer issues certain supplements and corrections with respect to the traditional version of the assumed theory, for instance, for the FSDT.

## References

[1] Reissner E. (1944). On the theory of bending of elastic plates. J. Math. Phys..

[2] Reissner E. (1945). The effect of transverse shear deformation on the bending of elastic plates. ASME J. Appl. Mech..

[3] Mindlin R.D. (1951). Influence inertia and shear on flexural motions of isotropic, elastic plates. ASME J. Appl. Mech..

[4] Baptista M. (2010). An elementary derivation of basic equations of the Reissner and Mindlin plate theories. Eng. Struct..

[5] Baptista M. (2011). Refined Mindlin-Reissner theory of forced vibrations of shear deformable plates. Eng. Struct..

[6] Baptista M. (2012). Comparison of Reissner, Mindlin and Reddy plate models with exact three dimensional solution for simply supported isotropic and transverse inextensible rectangular plate. Meccanica.

[7] Labuschagne A., van Resenburg N.F.J., van der Merwe A.J. (2009). Vibration of a Reissner-Mindlin-Timoshenko plate beam system. Math. Comput. Model..

[8] Wang C.M., Lim G.T., Reddy J.N., Lee K.H. (2001). Relationships between bending solutions of Reissner and Mindlin plate theories. Eng. Struct..

[9] Chróścielewski J., Makowski J., Pietraszkiewicz W. (2004). Statyka i Dynamika Powłok Wielopłatowych.

[10] Woźniak C. (2001). Mechanics of Elastic Plates and Shells.

[11] Wu S.R. (2004). Reissner-Mindlin plate theory for elastodynamics. J. Appl. Math..

[12] Lo K.H., Christensen R.M., Wu E.M. (1977). A high-order theory of plate deformation, Part 1: Homogeneous plates. ASME J. Appl. Mech..

[13] Lo K.H., Christensen R.M., Wu E.M. (1977). A high-order theory of plate deformation. Part 2: Laminated plates. ASME J. Appl. Mech..

[14] Reddy J.N. (1990). A general non-linear third-order theory of plates with moderate thickness. Int. J. Non-Linear Mech..

[15] Reddy J.N., Phan N.D. (1985). Stability and vibration of isotropic, orthotropic and laminated plates according to a higher-order shear deformation theory. J. Sound Vib..

[16] Volmir A.S. (1956). Flexible Plates and Shells.

[17] Cai L., Rong T., Chen D. (2002). Generalized mixed variational methods for Reissner plate and its applications. Comput. Mech..

[18] Cen S., Shang Y. (2015). Developments of Mindlin-Reissner plate elements. Math. Probl. Eng..

[19] Endo M., Kimura N. (2007). An alternative formulation of the boundary value problem for the Timoshenko beam and Mindlin plate. J. Sound Vib..

[20] Ghugal Y.M., Shimpi R.P. (2002). A review of refined shear deformation theories of isotropic and anisotropic laminated plates. J. Reinf. Plast. Compos..

[21] Kim S.E., Thai H.-T., Lee J. (2009). A two variable refined plate theory for laminated composite plates. Compos. Struct..

[22] Kim S.-E., Thai H.-T., Lee J. (2009). Buckling analysis of plates using the two variable refined plate theory. Thin-Walled Struct..

[23] Nelson R.B., Lorch D.R. (1974). A refined theory for laminated orthotropic plates. J. Appl. Mech..

[24] Park M., Choi D.-H. (2018). A two-variable first-order shear deformation theory considering in-plane rotation for bending, buckling and free vibration analyses of isotropic plates. Appl. Math. Model..

[25] Reddy J.N. (1984). A refined nonlinear theory of plates with transverse shear deformation. Int. J. Solids Struct..

[26] Shimpi R.P., Patel H.G. (2006). A two variable refined plate theory for orthotropic plate analysis. Int. J. Solids Struct..

[27] Shimpi R.P., Patel H.G. (2006). Free vibrations of plate using two variable refined plate theory. J. Sound Vib..

[28] Shimpi R.P. (2002). Refined plate theory and its variants. AIAA J..

[29] Soldatos K.P. (1988). On certain refined theories for plate bending. ASME J. Appl. Mech..

[30] Thai H.-T., Kim S.-E. (2010). Free vibration of laminated composite plates using two variable refined plate theory. Int. J. Mech. Sci..

[31] Shimpi R.P., Shetty R.A., Guha A. (2017). A single variable refined theory for free vibrations of a plate using inertia related terms in displacements. Eur. J. Mech. A Solids.

[32] Vasiliev V.V. (2000). Modern conceptions of plate theory. Compos. Struct..

[33] Taylor M.W., Vasiliev V.V., Dillard D.A. (1997). On the problem of shear-locking in finite elements based on shear deformable plate theory. Int. J. Solids Struct..

[34] Vasiliev V.V. (1992). The theory of thin plates. Mech. Solids.

[35] Vasiliev V.V. (1995). A discussion on classical plate theory. Mech. Solids.

[36] Vasiliev V.V., Lure S.A. (1992). On refined theories of beams, plates, and shells. J. Comput. Math..

[37] Allman D.J. (1984). A compatible triangular element including vertex rotations for plane elasticity analysis. Comput. Struct..

[38] Averill R.C., Reddy J.N. (1990). Behaviour of plate elements based on the first order shear deformation theory. Eng. Comput..

[39] Bathe K.J. (1996). Finite Element Procedures.

[40] Kant T. (1982). Numerical analysis of thick plates. Comput. Methods Appl. Mech. Eng..

[41] Zienkiewicz O.C., Taylor R.L. (1991). The Finite Element Method.

[42] Jones R.M. (1999). Mechanics of Composite Materials.

[43] Thai H.-T., Choi D.-H. (2013). A simple first-order shear deformation theory for laminated composite plates. Compos. Struct..

[44] Manevich A., Kolakowski Z. (2014). To the theory of transverse vibration of plates regarding shear deformation. Int. Appl. Mech..

[45] Bilstein W. (1974). Anwendung der Nichtlinearen Beultheorie auf Vorverformte, Mit Diskreten Laengssteifen Verstaerkte Rechteckplatten unter Laengsbelastung.

[46] Kolakowski Z., Krolak M. (2006). Modal coupled instabilities of thin-walled composite plate and shell structures. Compos. Struct..

[47] (2019). User’s Guide ANSYS^®^.

